# Characterization of radiographic features of consecutive lumbar spondylolisthesis

**DOI:** 10.1097/MD.0000000000005323

**Published:** 2016-11-18

**Authors:** Yapeng Sun, Hui Wang, Dalong Yang, Nan Zhang, Sidong Yang, Wei Zhang, Wenyuan Ding

**Affiliations:** Department of Spine Surgery, the Third Hospital of Hebei Medical University, Shijiazhuang, Hebei Province, China.

**Keywords:** angular displacement, consecutive lumbar spondylolisthesis, pelvic sagittal parameters, Taillard index

## Abstract

Radiographic features of consecutive lumbar spondylolisthesis were retrospectively analyzed in a total of 17 patients treated for this condition at the Third Hospital of Hebei Medical University from June 2005 to March 2012.

To investigate the radiographic features, pelvic compensatory mechanisms, and possible underlying etiologies of consecutive lumbar spondylolisthesis.

To the best of our knowledge, there is no previous report concerning the characteristics of consecutive lumbar spondylolisthesis.

The Taillard index and the lumbar lordosis (LL), pelvic incidence (PI), sacrum slope (SS), and pelvic tilt (PT) were determined on lateral X-ray images, and the angular displacement was analyzed on flexion–extension X-ray images. Correlation between LL and various pelvic parameters and correlation between Taillard index and angular displacement were assessed by Pearson correlation analysis.

A total of 20 cases of isthmic spondylolisthesis and 14 of degenerative spondylolisthesis were retrospectively studied in 17 patients. The Taillard index and the angular displacement in the lower vertebrae were both larger than those in the upper vertebrae. Statistical analysis revealed that LL was correlated with PI and PT, whereas PI was correlated with PT and SS. However, no correlation was identified between Taillard index and angular displacement.

In consecutive lumbar spondylolisthesis, the degree of vertebral slip and the angular displacement of the lower vertebrae were both greater than those of the upper vertebrae, indicating that the compensatory mechanism of the pelvis plays an important role in maintaining sagittal balance.

## 1 Introduction

Lumbar spondylolisthesis is a common condition treated by spinal surgery.^[1–3]^ It is usually due to congenital dysplasia, trauma, strain, or other causes of abnormalities in the bony connection between adjacent vertebrae, leading to partial or complete slippage of one vertebrae on adjacent vertebrae.^[4,5]^ It is noteworthy that it rarely causes unilateral pedicle stress fracture. The typical symptoms of this condition are neurological deficits, including low back pain, nerve root irritation, and neural dysfunctions. The most common types of this entity are isthmic and degenerative spondylolisthesis. A variety of surgical fusion techniques, such as anterior interbody fusion, posterior interbody fusion, posterolateral fusion, repair of the pars interarticularis, and reduction and fusion have been applied to stabilize the spine, relieve pain, and improve the patients’ life quality.^[6,7]^

Spondylolisthesis is most common in middle-aged women, affecting mainly L4–L5.^[8–10]^ Etiologies of spondylolisthesis include bilateral spondylolisthesis, spondylolysis, and interbody chronic dislocation. Radiological investigation is the key component of evaluation of lumbar spondylolisthesis to determine its anatomical abnormalities, etiologies, severity, and possible pathogenic mechanisms to guide the clinical management and assess the prognosis. For this purpose, a number of X-ray, CT, and MRI techniques have been employed to analyze anatomy of vertebrae, lumbar lordosis (LL), and the facet joints associated with the occurrence of slippage.^[11–14]^ In contrast to the abundant radiological data on spondylolisthesis of single vertebral bodies, data for consecutive lumbar spondylolisthesis are absent, although multilevel lumbar spondylolisthesis does occur and accounts for up to 11% of spondylolisthesis. Importantly, multilevel segmental involvement is of considerable significance for the occurrence of cauda equina syndrome.^[15]^

Herein, we report our results on the radiological features of consecutive spondylolisthesis. We identified a correlation between the forward displacement of the involved vertebrae and pelvic sagittal parameters. Our findings suggest that pelvic compensatory mechanisms play a role in maintaining the overall sagittal spinal and pelvic stability.

## 2 Materials and methods

### 2.1 Study subjects

A total of 967 patients were diagnosed with and treated for spondylolisthesis at our hospital from June 2005 to March 2012. Among them, 17 consecutive spondylolisthesis cases (1.75%) were identified in 5 males and 12 females with a median age of 56 years. Out of these 17 patients, 7 had spondylolisthesis involving L3–L4, and 10 had spondylolisthesis involving L4–L5.

The enrollment criteria for these patients were as follows: No spinal fractures, or scoliosis history; no history of spinal surgery; diagnosis of consecutive spondylolisthesis on a lateral lumbar spine X-ray.

### 2.2 Radiological assessment

**Figure 1:**
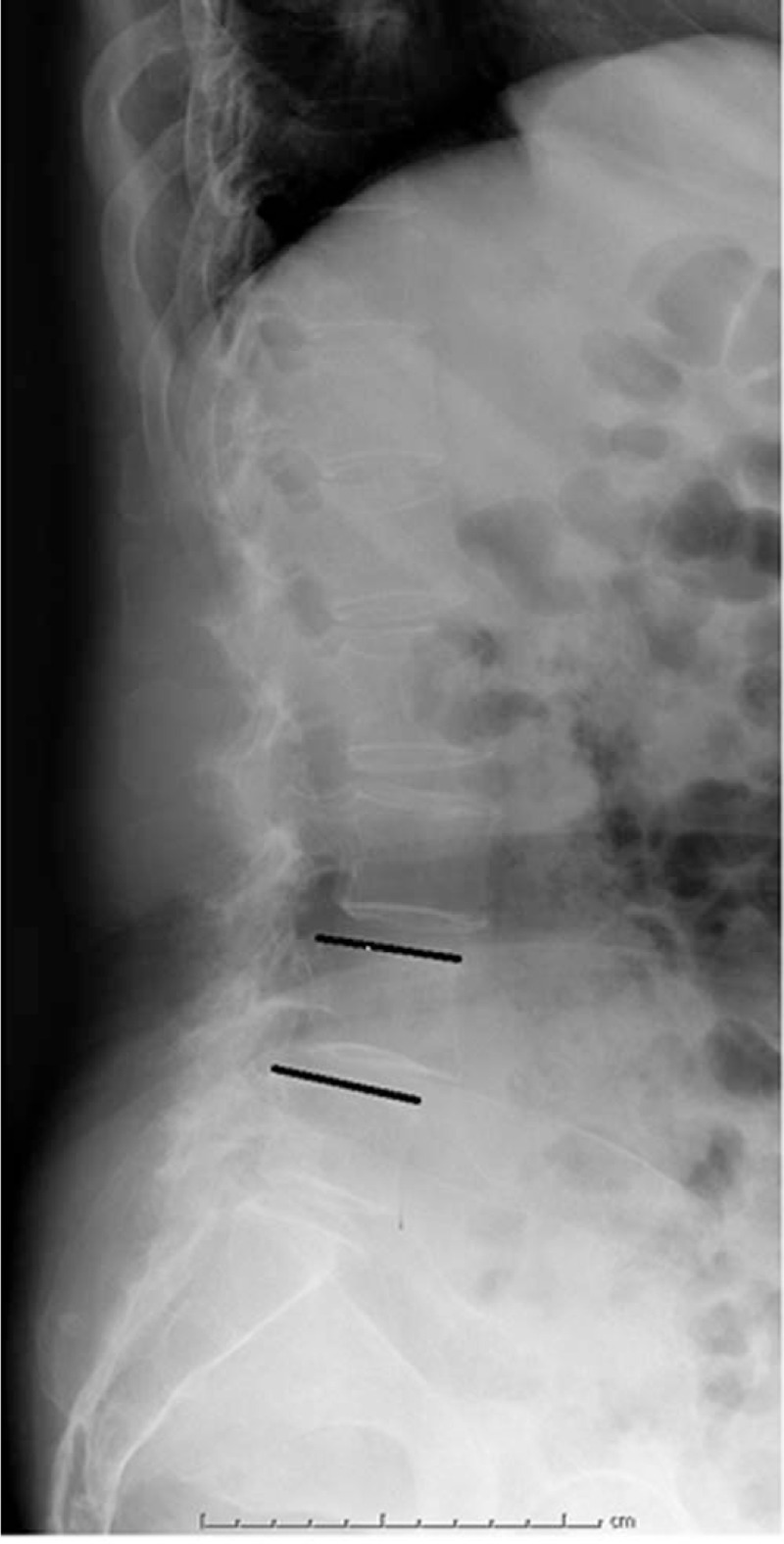
The measurement of Taillard index: the forward displacement distance of upper vertebral body/the length of the upper vertebral body × 100%.

**Figure 2:**
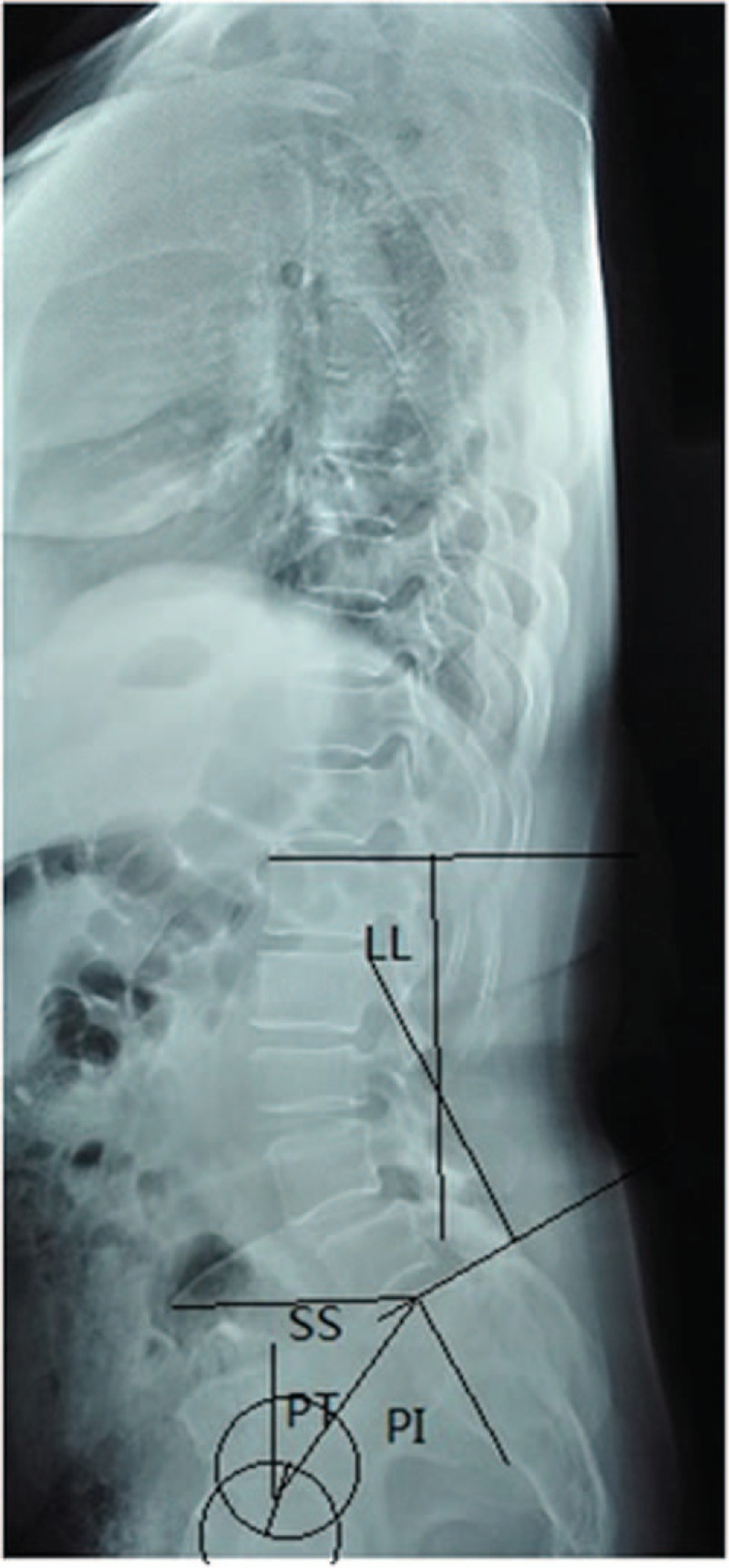
The measurement of the lumbar lordosis (LL), pelvic incidence (PI), pelvic tilt (PT), and sacrum slope (SS).

**Figure 3:**
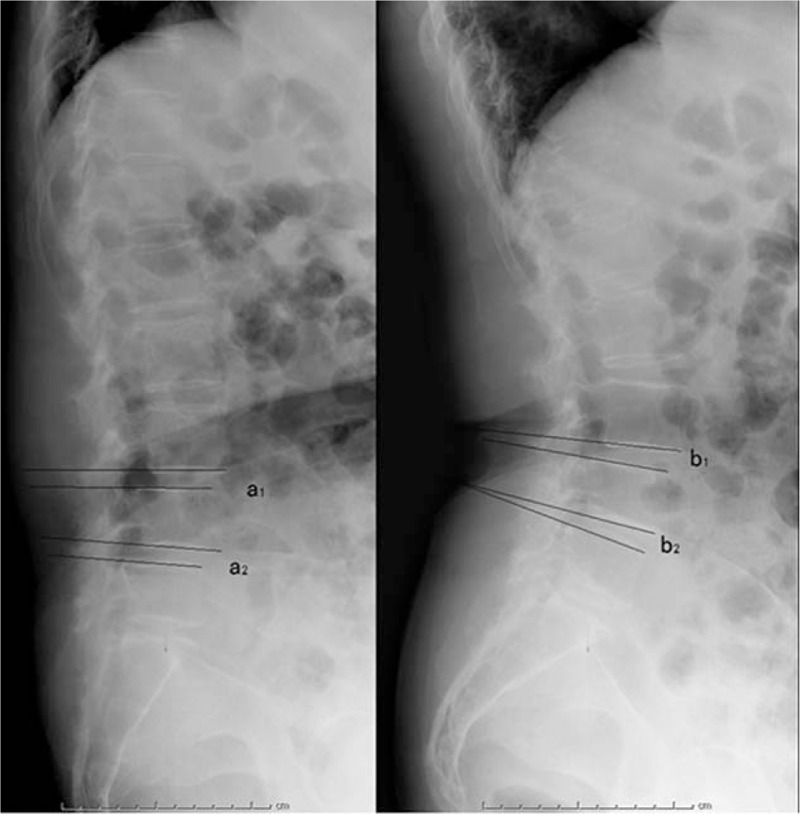
The measurement of the angular displacement on flexion–extension X-ray. The angular displacement of the upper intervertebral space was calculated as (a_1_–b_1_); the angular displacement of lower intervertebral space was determined by calculating (a_2_–b_2_).

The lateral X-ray images for individual patients were retrieved from our Picture Archiving and Communication Systems (PACS). The Taillard index was defined as the relative displacement distance between the involved vertebrae divided by the horizontal length of the upper vertebral body (Fig. 1). The LL was the angle between the L1 endplate and S1 endplate. The pelvic incidence (PI) was denoted as the angle between the vertical line of S1 endplate and the line connecting midpoint of S1 endplate to midpoint of the femoral heads. The sacrum slope (SS) was defined as the angle between the S1 endplate and the horizontal line, while the pelvic tilt (PT) was the angle formed by the vertical line and the line connecting the midpoint of S1 endplate to the midpoint of the femoral heads (Fig. 2). The displacement between the upper and lower intervertebral space was determined on flexion–extension dynamic X-ray radiographs (Fig. 3).

### 2.3 Statistical analysis

The data were analyzed with SPSS13.0. Student *t* test was used to analyze the relative anterior displacement of the vertebrae and the angular displacement of the intervertebral spaces. Pearson correlation analysis was applied to investigate the correlation between LL and pelvic sagittal parameters and the correlation between Taillard index and angular displacements. *P* < 0.05 was considered statistically significant.

### 2.4 Ethical review

This study was approved by the Ethical Committee of the Third Hospital of Hebei Medical University, Shijiazhuang, Hebei, China.

## 3 Results

Twenty isthmic (upper and lower vertebrae) and 14 (upper and lower vertebrae) degenerative consecutive spondylolisthesis were identified in 34 vertebral bodies in 17 patients (Table 1). Among the above mentioned 7 patients with L3–L4 consecutive spondylolisthesis. Out of the 10 patients with consecutive spondylolisthesis, 7 were with isthmic and 3 with degenerative spondylolisthesis (Fig. 1).

**Table 1:**
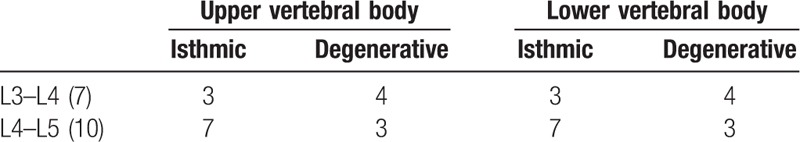
The type of lumbar spondylolisthesis.

The average Taillard index of the upper vertebrae was 17.6 ± 4.1%, whereas its value for the lower vertebrae was 22.4 ± 4.1% (*t* = 7.672, *P* < 0.001). The average angular displacement of the upper vertebrae was 10.8 ± 2.6°, whereas that of the lower vertebrae was 18.6 ± 5.5° (*t* = 5.251, *P* < 0.001).

In the 17 patients, the average LL was 58.1 ± 4.3°, the average PI 68.7 ± 4.8°, the average SS 37.2 ± 3.8°, and the average PT 31.6 ± 4.0°. A correlation between LL and PI and PT was identified, while PI was correlated with PT and SS (Table 2). In contrast, no correlation was present between Taillard index and the angular displacement (*P* > 0.05).

**Table 2:**
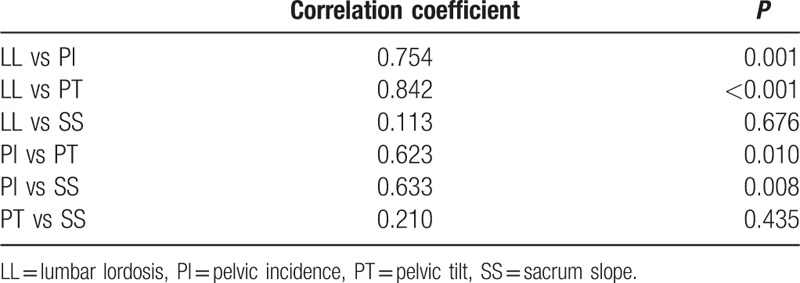
Correlation between LL and the pelvic parameters.

## 4 Discussion

Spondylolisthesis has a range of causes. For example, isthmus crack spondylolisthesis can be due to a congenital isthmus defect, an acute lumbar trauma leading to an isthmus fracture, stress-related fractures caused by chronic fatigue on the basis of congenital isthmus dysplasia. This condition is commonly detected in the 5th lumbar vertebral body in 30- to 40-year-old adults, as males and females are affected approximately equally. On the other hand, degenerative spondylolisthesis is usually secondary to intervertebral disc degeneration and commonly affects L4–L5 in 50- to 60-year-old women.^[4,5,10]^ Although the diverse causative factors usually lead to single-level spondylolisthesis, we found that consecutive spondylolisthesis is not a rare condition, especially in individuals performing long-term heavy physical labor, such as the subjects enrolled in the present study. We also discovered that pelvic compensation mechanisms play a key role in maintaining the overall sagittal spinal and pelvic stability in this condition. To the best of our knowledge, this is the first radiographic report regarding the biomechanical features of consecutive spondylolisthesis.

Due to its distinct anatomical nature, isthmic spondylolisthesis is considered to be inherently more instable than degenerative spondylolisthesis, a fact reflected by the difference in the angular displacement existing between these 2 types.^[15–17]^ In the present study, identical types of spondylolisthesis in the 2 levels of vertebrae were present in each individual. Therefore, we believe that the effects exerted by the type of spondylolisthesis on angle displacement were minimal in the studied patients.

Interbody gravity force is transmitted and divided into a compression force perpendicular to the shift force that is parallel to the vertebral endplates.^[18]^ In general, lower vertebral bodies bear greater shearing forces with respect to the upper vertebrae, which can accelerate intervertebral degeneration in the disc and facet joints. Consequently, from an anatomical standpoint, the range of movement in the discs that are closer to the lumbosacral region is greater than that in those located away from the lumbosacral region. In line with this mechanism, we found that the angular displacement in the lower vertebral bodies was much more prominent than that in the upper vertebrae in the clinical setting of consecutive spondylolisthesis.

It is worth noting that the antishearing force mechanisms are compromised in lumbar spondylolisthesis.^[19–21]^ To compensate for this defect, a series of compensatory changes are realized in the spine–pelvis sequence to adapt and maintain a stable standing position.^[22–24]^ These adaptive changes are usually reflected by the alteration of spine–pelvis sagittal parameters, such as PI. A number of studies have shown that the extent of increase of PI values in patients with single-level lumbar spondylolisthesis is positively correlated with the severity of displacement of the involved vertebrae.^[21,24]^ Barrey et al^[25]^ has further suggested that PI value is to some extent predictive for displacement of affected vertebrae in 1-level spondylolisthesis. We calculated the PI values in 2-level spondylolisthesis and found that the average PI value in consecutive spondylolisthesis was higher than the reported ones in single-level segment spondylolisthesis (68.7° vs 66.3°),^[26]^ and not surprisingly either, higher than that in healthy individuals.^[26,27]^ We postulated that the high PI values in consecutive spondylolisthesis might be a risk factor for displacement of the affected vertebrae. Nevertheless, to evaluate the biomechanical relevance of an increased PI value in spondylolisthesis requires further prospective studies.

Berthonnaud et al^[28]^ proposed the hinged concept of connection from head to pelvis. Any change in orientation within an anatomical spine segment will cause adaptive changes in adjacent segments in order to maintain the stability of the body. For example, Vialle et al^[29]^ have reported that SS values in single-vertebral lumbar spondylolisthesis gradually increase along with the degree of displacement in spondylolisthesis grade I–III, but decrease in grades IV–V. Although our results clearly demonstrated that consecutive spondylolisthesis resulted in a modification of pelvic parameters, our data indicated that the values of SS (37.2°) and PT (31.6°) in patients with consecutive spondylolisthesis were higher than those observed in the healthy population (PT = 25.1° and SS = 30.7°)^[29–31]^ in the same age group. In the present study, all the Taillard indexes were below 50%, classifying the grade of spondylolisthesis as I or II. We hypothesize, that the compensatory mechanisms in the spine–pelvis sequence in 2-segment spondylolisthesis might be different from that in 1-level spondylolisthesis. Another set of data supporting this notion was the failure of identification of the existence of a significant correlation between PT and SS, which was compatible with the reported finding indicating that PI = SS + PT, and there are opposite trend shifts of PT and SS in spondylolisthesis.^[32–34]^ This discrepancy could be due to two reasons. First, consecutive spondylolisthesis may cause a distinct pelvic–spine compensation which leads to a discordant change in PT and SS. Second, the small sample size and selection bias in the present study might have led to the occurrence of this discrepancy.

As a limitation of the current investigation, we have to point out that the data collected in the present study are derived from X-ray images only. Since whole-spine imaging was not employed, we were unable to conduct an analysis of the whole-spine sequence.

In summary, our study showed that in the spondylolisthesis of 2 adjacent lumbar segments both the degree of the vertebral slip and the angular displacement of the lower vertebrae were greater, than those of the upper vertebrae, indicating that the compensatory mechanism of the pelvis plays an important role in maintaining the sagittal balance.
